# IspE Inhibitors Identified by a Combination of *In Silico* and *In Vitro* High-Throughput Screening

**DOI:** 10.1371/journal.pone.0035792

**Published:** 2012-04-25

**Authors:** Naomi Tidten-Luksch, Raffaella Grimaldi, Leah S. Torrie, Julie A. Frearson, William N. Hunter, Ruth Brenk

**Affiliations:** Division of Biological Chemistry and Drug Discovery, College of Life Sciences, University of Dundee, Dundee, United Kingdom; Spanish National Cancer Center, Spain

## Abstract

CDP-ME kinase (IspE) contributes to the non-mevalonate or deoxy-xylulose phosphate (DOXP) pathway for isoprenoid precursor biosynthesis found in many species of bacteria and apicomplexan parasites. IspE has been shown to be essential by genetic methods and since it is absent from humans it constitutes a promising target for antimicrobial drug development. Using *in silico* screening directed against the substrate binding site and *in vitro* high-throughput screening directed against both, the substrate and co-factor binding sites, non-substrate-like IspE inhibitors have been discovered and structure-activity relationships were derived. The best inhibitors in each series have high ligand efficiencies and favourable physico-chemical properties rendering them promising starting points for drug discovery. Putative binding modes of the ligands were suggested which are consistent with established structure-activity relationships. The applied screening methods were complementary in discovering hit compounds, and a comparison of both approaches highlights their strengths and weaknesses. It is noteworthy that compounds identified by virtual screening methods provided the controls for the biochemical screens.

## Introduction

Isoprenoids constitute one of the largest groups of natural product compounds. They are structurally diverse and include cannabinoids, essential oils, sterols, the prenyl groups of chlorophyll and RNA among others. Isoprenoids are involved in respiration, hormone-based signalling, the post-translational processes that control lipid biosynthesis, meiosis, apoptosis, glycoprotein biosynthesis, and protein degradation. Furthermore, they represent important structural components of cell membranes [1], [2], [3].

**Figure 1:**
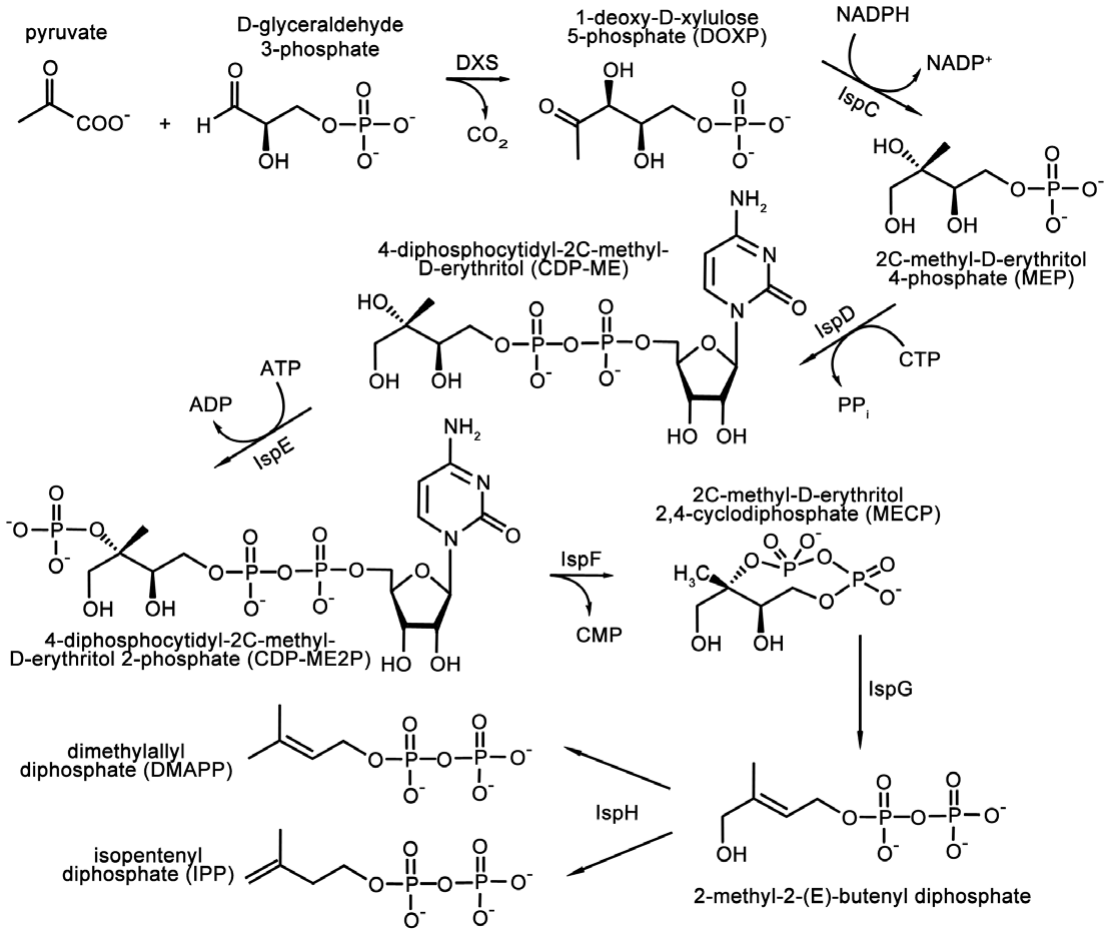
Non-mevalonate pathway providing the isoprenoid precursors IPP and DMAPP.

All isoprenoids are synthesised from two simple precursors, isopentenyl pyrophosphate (IPP) and dimethylallyl pyrophosphate (DMAPP). The precursors are provided by two distinct biosynthetic pathways, which are distributed in an organism specific manner. In mammals, the plant cytosol, certain bacteria and trypanosomatids, these compounds are products of the mevalonate (MVA) pathway. In most eubacteria, algae, chloroplasts, cyanobacteria and apicomplexan parasites the deoxy-xylulose phosphate (DOXP) pathway (also called the non-mevalonate pathway) generates IPP and DMAPP (Figure 1) [4], [5], [6], [7].

This biosynthetic route to isoprenoid precursors is an essential aspect of metabolism and the DOXP pathway is a genetically validated target for broad-spectrum antimicrobial drugs against malaria, tuberculosis, and a range of sexually transmitted conditions [8]. The absence of this pathway in humans makes it a particular attractive target for antimicrobial drug discovery. Chemical validation is provided by the anti-malarial compound fosmidomycin, which inhibits 1-deoxy-D-xylulose 5-phosphate reductoisomerase (IspC, Figure 1) [9]. We have turned our attention to another enzyme in the pathway, 4-diphosphocytidyl-2C-methyl-D-erythritol (CDP-ME) kinase (IspE, Figure 1).

**Figure 2:**
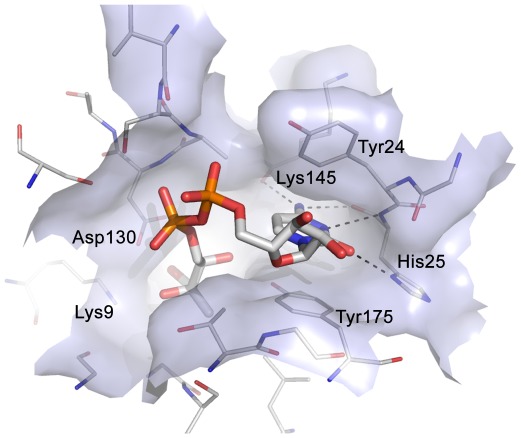
Substrate binding site of *Aa*IspE (PDB code 2v2z). The catalytic residues Lys9 and Asp130 are labelled together with other residues important for ligand binding. The cytidine moiety of the substrate forms hydrogen bonds with Lys145 and His25, π-stacking interactions with Tyr175 and edge-face interactions with Tyr24.

**Figure 3:**
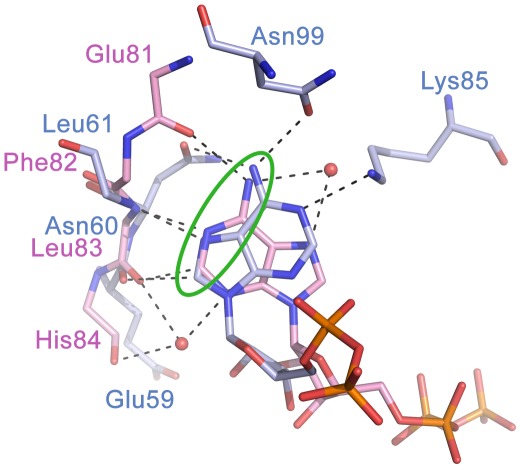
Superposition of adenine binding sites of human CDK2 and *Aa*IspE. ATP adopts the *anti* conformation in *h*CDK2 (PDB code 2cch, carbon atoms coloured pink) while the ATP analogue AMP-PNP adopts the *syn* conformation in *Aa*IspE (PDB code 2v8p, carbon atoms coloured light blue). Further, in *h*CDK2 adenine forms hydrogen bonds with the backbone amide groups of the hinge region while in *Aa*IspE the backbone and side chain atoms of surrounding amino acids are involved in hydrogen bonding-interactions. In both enzymes, a donor-acceptor-donor motive (green circle) is important for molecular recognition.

IspE catalyses the transfer of the ATP γ-phosphate to 4-diphosphocytidyl-2C-methyl-d-erythritol (CDP-ME) forming 4-diphosphocytidyl-2C-methyl-d-erythritol 2-phosphate (CDP-ME2P) and ADP. The gene encoding IspE has been shown to be essential for survival in *Escherichia coli*, *Bacillus subtilis*, *Haemophilus influenzae*, and *Mycobacterium tuberculosis* [10], [11], [12], [13], [14], [15]. Crystal structures of IspE from *Aquifex aeolicus* (*Aa*IspE), *E. coli*, *Thermus thermophilus* and *M. tuberculosis* have been determined [16], [17], [18], [19], [20], [21]. Our recent work has concentrated on *Aa*IspE since it is a soluble, stable enzyme for which reproducible protein crystals can be obtained [16], [17], [18]. *Aa*IspE has been co-crystallised with substrate and a non-hydrolysable ATP derivative (AMP-PNP) and also inhibitors that are structurally related to the substrate [18]. The cytidine moiety of the substrate binds in a well-defined pocket and forms hydrogen bonds with Lys145 and His25, π-stacking interactions with Tyr175 and edge-face interactions with Tyr24 (*Aa*IspE numbering, Figure 2). The binding of the co-factor by IspE is unusual. Generally, ATP binds to kinases with the purine moiety in *anti* conformation with respect to the ribose. In contrast, in IspE, the energetically less favourable *syn* conformation was found (Figure 3). Further, in a typical protein kinase pocket the adenine moiety forms hydrogen bonds with the backbone amide group of the so called hinge region via N1, C2, and the exocyclic amino group [22]. In IspE, it is N1, N7, C8 and the exocyclic amino group that are involved in hydrogen-bonds with surrounding amino acids. Despite these differences, the typical donor–acceptor–donor motif found in protein kinase inhibitors is still present in IspE (Figure 3).

**Figure 4:**
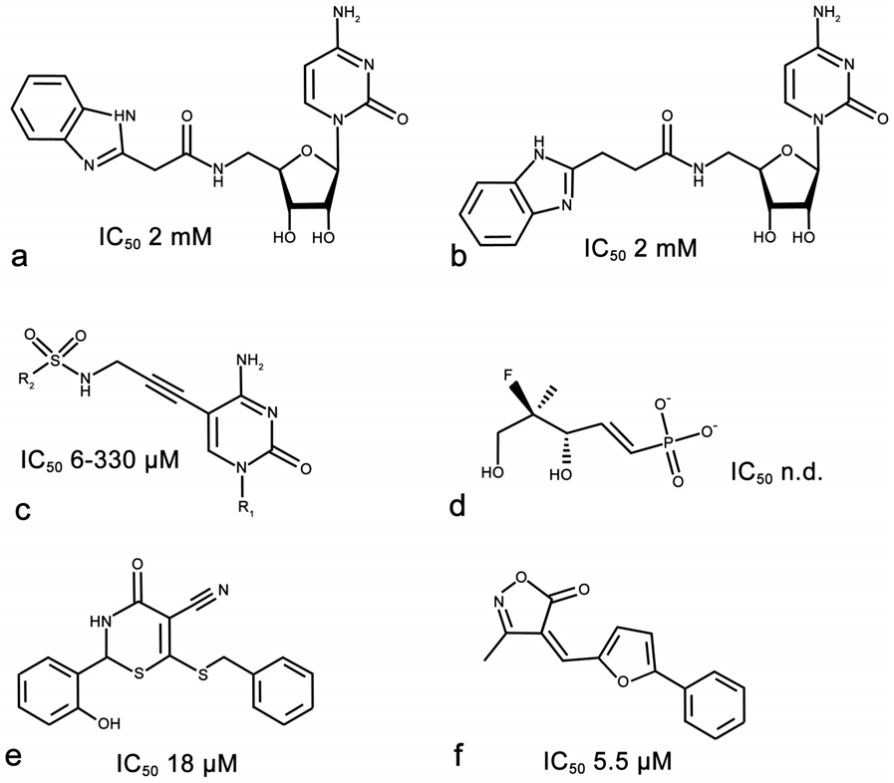
Substrate-like (a–d) and non-substrate-like (e–f) IspE inhibitors together with inhibition values [**16]**, [17], [18], [23], [24****].

Two types of IspE inhibitors are known. The majority of IspE inhibitors mimic either the cytidine or phosphate-sugar moiety of the substrate CDP-ME (Figure 4a–d) [16], [17], [18], [23], [24]. Crystal structures of *Aa*IspE in complex with cytidine analogues containing a benzimidazole moiety attached to the ribose (Figure 4a and b) have been determined suggesting that interactions in the cytidine pocket are key for binding affinity [16]. Considering the size of these molecules they are rather weak ligands for *Ec*IspE with affinities in the double-digit micromolar range. In contrast, the smaller cytosine analogues (Figure 4c) bind more tightly to *Ec*IspE with some compounds of this series displaying IC_50_ values in the low micromolar range [17], [23]. Very recently, non-substrate like *Ec*IspE inhibitors have been reported [24]. The best characterized compounds also have IC_50_ values in the low micromolar range (Figure 4e and f). They were proposed to bind into the substrate binding site forming stacking interactions with Tyr25 and Phe185 (Tyr24 and Tyr175 in *Aa*IspE, Figure 2); however, the derived structure-activity relationships (SAR) were not always consistent with this binding mode as large changes to the presumably pi-stacking moieties did not lead to large changes in affinity.

Motivated by the potential of IspE as a target for broad-spectrum antimicrobial drugs we sought to discover non-substrate like IspE inhibitors that can serve as starting points for the development of new antimicrobials. There are several methods for hit discovery. They can be divided into *in silico* and *in vitro* approaches. [25], [26], [27]. Using both approaches, either lead-like or fragment-like libraries can be screened. Lead-like libraries typically deliver fewer but more potent hits compared to screening smaller, fragment-like compounds which often leads to a higher hit rate albeit frequently associated with weaker binding. If the structure of the target is known, molecular docking is a viable *in silico* method [28]. There are several studies that compare the outcomes of docking and *in vitro* high-throughput screening [29], [30], [31], [32], [33], [34], [35], [36], [37], [38]. These studies suggest that often the two methods identify different hit compounds. Reasons for this are that as a result of virtual screening usually only few compounds are tested experimentally which allows more robust assays to be used and testing at higher concentrations which can identify weaker inhibitors [29], [31], [32]. Further, much larger libraries can be screened computationally than it is affordable to screen biochemically [37]. On the other hand, due to shortcomings in docking algorithms and scoring functions, potential hits might be missed when only relying on computational methods [32], [35], [37], [38]. To benefit from the advantageous of these complementary strategies, we decided to apply both for hit discovery for IspE.

The substrate and co-factor binding sites of IspE are highly conserved across difference species. [16], [18]. Therefore, in principle, given the high level of conservation in IspE across species either structure could serve as a template for structure-based design of inhibitors with broad-spectrum antimicrobial activity. However, since we had been able to reproducibly crystallize and gain most crystallographic information with *Aa*IspE we decided to use the former for virtual screening. The intention was then to determine crystal structures of new inhibitors in complex with *Aa*IspE. As *A. aeolicus* is a thermophilic organism with the optimal temperature of *Aa*IspE activity near 60°C [18] and working at such elevated temperatures is not practical for a biochemical screen, it was decided to use *E. coli* IspE (*Ec*IspE) for ligand binding characterisation. The high level of sequence conservation provided confidence in this approach [18].

Here, we report on our hit discovery efforts for IspE. The crystal structures were exploited for a structure-based ligand design approach leading to efficiently binding fragments likely addressing the cytidine-binding site. In addition, a biochemical screen of a focussed compound library was carried out resulting in two inhibitors with binding affinities in the low micromolar range. Hit compounds from both approaches were expanded to compound series. Compounds of these series have high ligand efficiencies [39] (≥0.29 kcal/mol per non-hydrogen atom) and possess favourable physico-chemical properties representing promising starting points for the synthesis of new IspE inhibitors. In addition, we compared the performance of *in silico* and *in vitro* screening and discuss their strengths and weaknesses.

## Results

### Virtual screening for IspE inhibitors

Analysis of *Aa*IspE crystal structures suggested that the cytidine moiety plays a key role in ligand binding (Figure 2) [16]. The cytidine binding site is formed by two aromatic amino acids (two tyrosines in *Aa*IspE and *Mt*IspE, of which one is replaced by phenylalanine in *Ec*IspE) which form stacking and edge-face interactions with the cytidine ring and a histidine residue that stabilizes ligand binding by forming hydrogen bonds with N3 and the exocyclic carbonyl and amino groups. This narrow cleft, promoting aromatic and polar interactions, appears well suited to accommodate small compounds based on scaffolds distinct from cytidine with potential to display high ligand efficiency.

**Figure 5:**
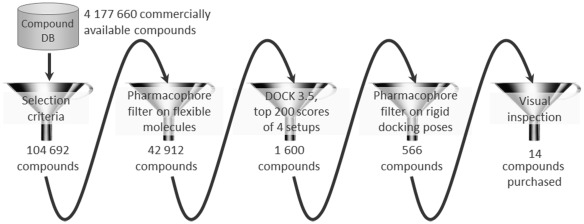
Virtual screening cascade used to identify potential IspE inhibitors together with number of compounds that passed each filter step.

A hierarchical screening strategy was adopted to retrieve fragments binding into the cytidine pocket of IspE (Figure 5). First, a database of commercially available compounds was filtered according to physico-chemical criteria. Next, a pharmacophore hypothesis was derived and used to screen all compounds passing the first filter step. The remaining compounds were docked into the *Aa*IspE binding site. Finally, the predicted binding modes were post-filtered and promising compounds were short-listed for purchase.

To derive a compound set for virtual screening, an in-house virtual library containing 4,177,660 commercially available compounds [40] was filtered by the following selection criteria: at least one but not more than five hydrogen-bond donors, at least one but not more than ten hydrogen-bond acceptors, at least nine but not more than 23 heavy atoms and a clogP between −1 and 4. In addition, the number of rotatable bonds was restricted to less than seven, the total charge between −1 and +1, and at least one but not more than two ring systems were allowed. Compounds containing unwanted (e.g. reactive or potentially toxic) functionalities were excluded. Only compounds that fulfilled all requirements (104,692) were taken to the next step.

**Figure 6:**
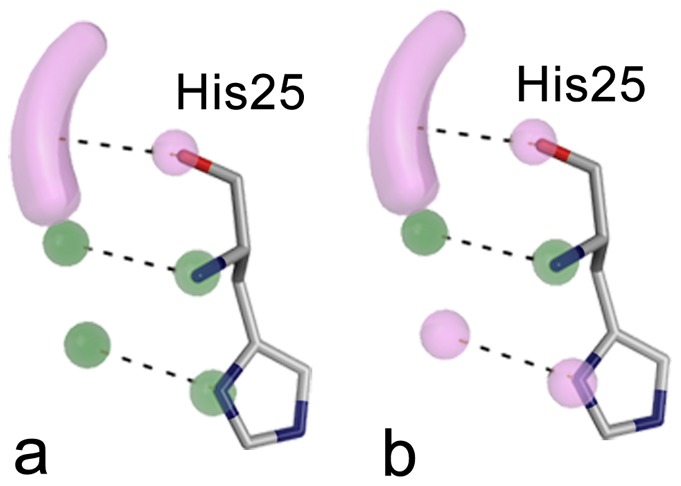
Structure-based pharmacophore for IspE inhibitors considering the possible tautomers of His25. Either ND (a) or NE (b) are carrying the hydrogen atom. Hydrogen-bond donor features and their corresponding binding partners in the protein are shown in purple and hydrogen-bond acceptor features and their corresponding binding partners are coloured green.

The selected subset was further filtered using a protein-based pharmacophore. When deriving the pharmacophore we aimed to strike a reasonable balance between a complex query which potentially retrieves very potent compounds but has only a very low hit rate and a relaxed query retrieving many compounds which prove not be active. To not be over descriptive we decided to only include interactions to His25 (*Aa*IspE numbering) which is essential for recognition of the cytosine moiety of the substrate. In all structures containing ligands interacting with this residue, ND presumably carries a hydrogen atom to hydrogen bond with the cytidine moiety of the ligands (Figure 2). However, in the crystal structures His25 NE is solvent exposed and not involved in a hydrogen-bonding network. Accordingly, it is possible that not ND but NE carries a hydrogen atom when challenged with ligands presenting a hydrogen-bond donor functionality. Therefore, both states were considered in the derived pharmacophore. Furthermore, hydrogen-bond acceptor interactions to the backbone amino group and hydrogen-bond donor interactions to the carbonyl group of His25 were required (Figure 6). 42,912 compounds fulfilled at least two of these pharmacophore features.

In the final step, the remaining compounds were docked into the receptor and promising hits selected for testing. For this purpose four different versions of the binding pocket were prepared taking into account different tautomers of His25 and the presence and absence of the co-factor. From each docking run, the top 200 scoring molecules together with the top 200 scoring molecules obtained when the score was divided by the number of heavy atoms were stored in the final hit list. The latter was done to favour small molecules which bind with a predicted high ligand efficiency [39]. The resulting 1,600 docking poses were filtered for compounds still in agreement with the described pharmacophore hypothesis (Figure 6). Only 566 compounds fulfilled at least two interactions required by the pharmacophore. By visual inspection compounds with additional hydrogen-bonding or hydrophobic interactions to the binding site were favoured and finally 14 compounds were purchased for evaluation. Five of these were predicted to have interactions with His25 similar to those observed for cytidine (Figure 6a) while nine compounds satisfied the alternative arrangement of functional groups (Figure 6b).

### Inhibition assays of shortlisted compounds

**Figure 7:**
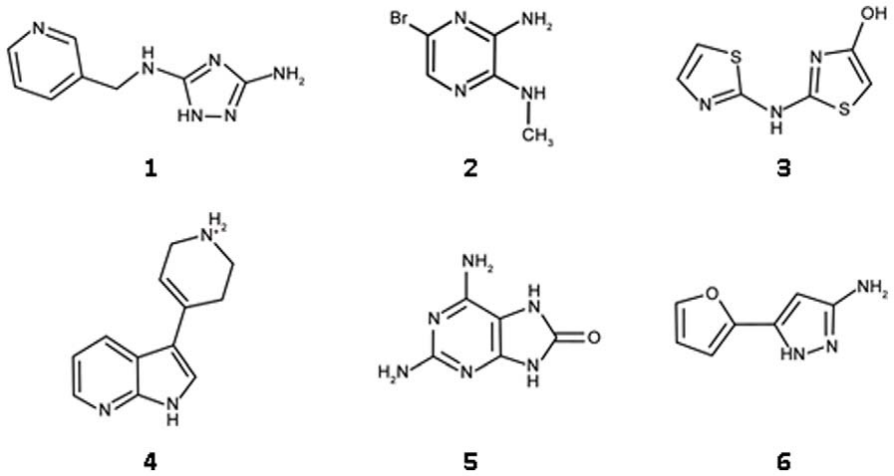
Chemical structures of virtual screening hits.

**Figure 8:**
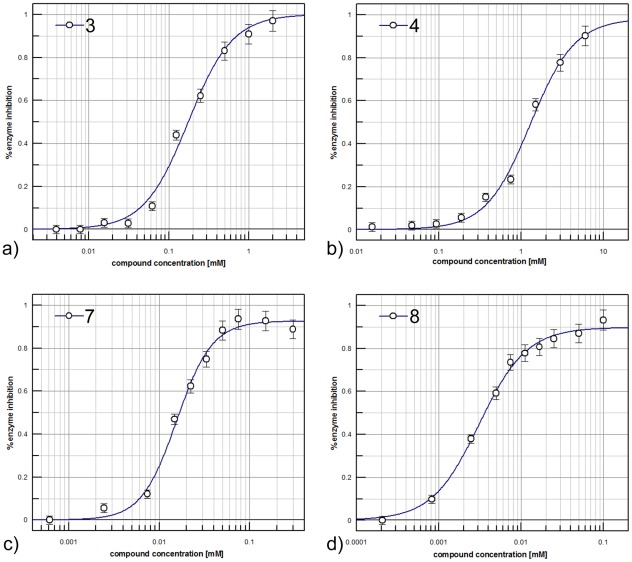
Potency curves of compounds 3 (a), 4 (b), 7 (c) and 8 (d).

As explained, access to a body of accurate structural information dictated that we use the *Aa*IspE structure for the virtual screening. However, *A. aeolicus* is a thermophilic organism and the optimal temperature of *Aa*IspE activity is near 60°C [18]. Therefore, we used *Ec*IspE for ligand binding characterisation. IC_50_ values could be determined for six compounds (Figure 7, Table 1, and Figure 8a and b,). Their IC_50_ values were in the high micromolar to low millimolar range with ligand efficiencies ranging from 0.28 to 0.54 kcal/mol per non-hydrogen atom. The most potent compound was **3** with an IC_50_ of 160 µM and a ligand efficiency of 0.50 kcal/mol per non-hydrogen atom.

**Table 1:** Docking ranks, physico-chemical properties, inhibition values, and ligand efficiencies for virtual screening hits.

ID	Rank_total^1^	MW [g/mol]	clogP	IC_50_ [M] (SD)^4^	Hill slope (SD)^4^	Ligand efficiency [kcal/mol per non-hydrogen atom]
	Rank_heav^2^					
	(Setup^3^)					
**1**	4849	190	0.10	1.8·10^−3^ (3·10^−4^)	1.6 (0.2)	0.33
	12					
	(4)					
**2**	19401	203	0.34	5.9·10^−4^ (3·10^−5^)	1.7 (0.3)	0.53
	3					
	(2)					
**3**	16081	199	1.92	1.6·10^−4^ (9·10^−6^)	1.0 (0.1)	0.50
	13					
	(3)					
**4**	74	200	1.22	1.5·10^−3^ (3·10^−4^)	1.3 (0.2)	0.32
	1					
	(4)					
**5**	12374	166	−0.57	2.3·10^−3^ (6·10^−5^)	2.6 (0.2)	0.37
	7					
	(3)					
**6**	16249	149	0.67	3.5·10^−3^ (4·10^−4^)	2.2 (0.5)	0.28
	7					
	(1)					

For chemical structures see Figure 7.

1 using the total score of docking for ranking (after application of the pharmacophore filter).

2 using the score divided by the number of heavy atoms of the molecule for ranking (after application of the pharmacophore filter).

3 setup 1: His25 protonated at ND, no ADP present; setup 2: His25 protonated at ND, ADP present; setup 3: His25 protonated at NE, no ADP present; setup 4: His25 protonated at NE, ADP present.

4 average values of three independent measurements, standard deviation in brackets.

### Biochemical screen of a focussed compound library

A focussed kinase-specific library consisting of 6,178 compounds was available to us [40]. All library compounds contain a scaffold capable of forming multiple hydrogen bonds with the hinge region of typical serine/threonine protein kinases which is an important recognition motif for ATP-competitive kinase inhibitors [22]. Despite structural differences between typical serine/threonine protein kinase adenine binding sites and the IpsE adenine binding site, both pockets require the same spatial distribution of hydrogen-bond donors and acceptors (Figure 3). Furthermore, one of the possible tautomers of the cytidine binding site is also consistent with this pharmacophore (Figure 6b). Therefore, screening this focussed kinase compound set seemed advantageous.

**Figure 9:**
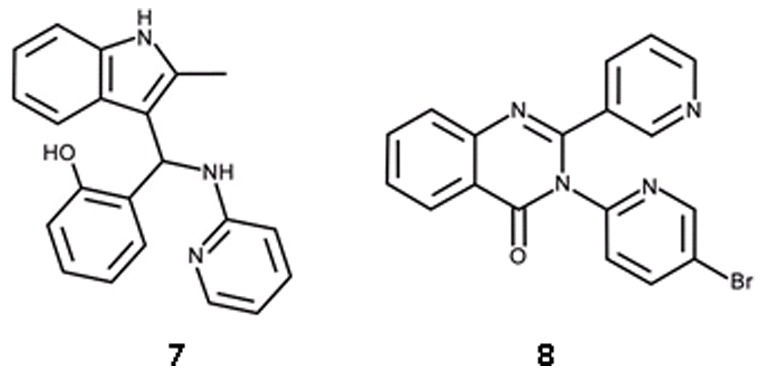
Chemical structures of *in vitro* screening hits.

The kinase library was screened in 384-well plates at 33 µM compound concentration. In order to provide a standard inhibitor for quality control a panel of typical protein kinase inhibitors, eg staurosporine, purvalanol and kenpaullone was evaluated but none of the compounds showed any *Ec*IspE inhibition at 100 µM. Therefore, compounds **3** and **4** (Table 1), which we identifed by virtual screening were used to monitor the assay performance. An average signal to noise ratio of 2.1 and an average Z′ value of 0.62 were obtained for the screen. Initial hits were re-assayed in duplicate at the same concentrations as used for the primary screen. This resulted in confirmed activity for two compounds (Figure 9, Table 2, and Figure 8c and d). The compounds were repurchased to determine their IC_50_ values. They inhibit *Ec*IspE in the low micromolar range (19 and 3 µM, respectively) and have ligand efficiencies comparable to the virtual screening hits (0.29 and 0.35 kcal/mol per non-hydrogen atom, respectively).

**Table 2:** Physico-chemical properties, inhibition values, and ligand efficiencies for *in vitro* screening hits.

ID	MW [g/mol]	clogP	IC_50_ [M] (SD)*	Hill slope (SD)*	Ligand efficiency [kcal/mol per non-hydrogen atom]
**7**	329	3.63	1.9·10^−5^ (2·10^−6^)	1.4 (0.1)	0.29
**8**	379	2.79	2.5·10^−6^ (4·10^−7^)	1.3 (0.1)	0.35

For chemical structures see Figure 9.

* average values of three independent measurements, standard deviation in brackets.

### Hit expansion and structure-activity relationships

Unfortunately, extensive co-crystallisation experiments and soaking of preformed *Aa*IspE crystals with the hits identified by the virtual and high-throughput screening approaches did not provide any structural information. Therefore, SAR for the virtual screening hits were derived based on the modelled binding modes. Only compounds with Hill coefficients close to one (**3** and **4** in Table 1) were followed up for hit expansion. Higher Hill coefficients are not consistent with a binding model for competitive binding to a single binding site and are possibly indicative of compound aggregation, solubility issues, an assay artefact, or more than a single class of binding sites in the assay solution [41], [42], [43]. The remaining compounds were therefore disregarded.

**Figure 10:**
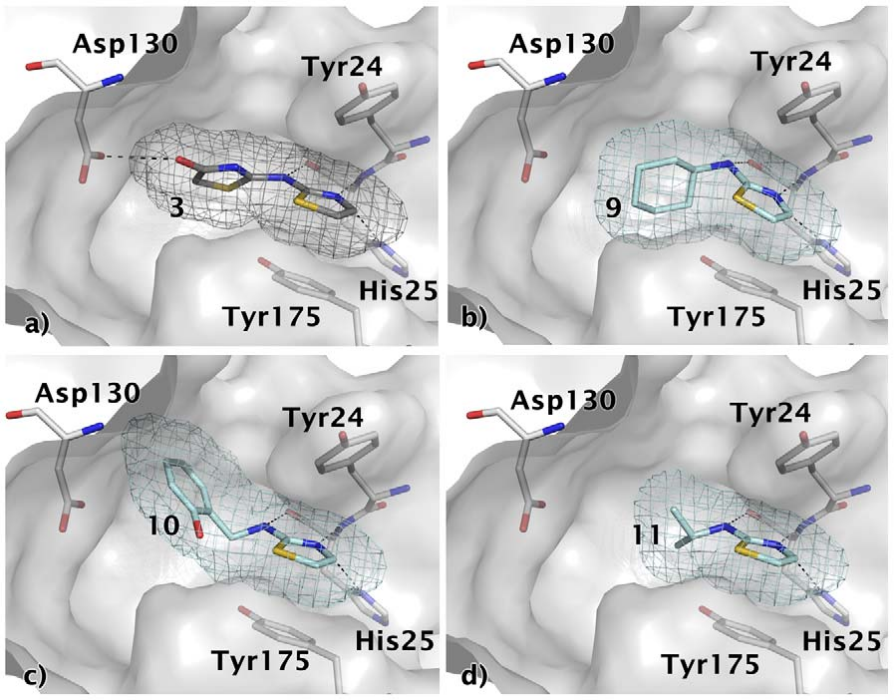
Modelled binding mode of compounds a) 3, b) 9, c) 10, and d) 11. The mesh represents the combined van der Waals radii of the ligand atoms. The aminothiazole core is predicted to be positioned between Tyr24 and Tyr175 and to form hydrogen bonds to His25. The virtual screening hit **3** is expected to form an additional hydrogen bond with Asp130, which none of its analogues is capable of doing.

**Figure 11:**
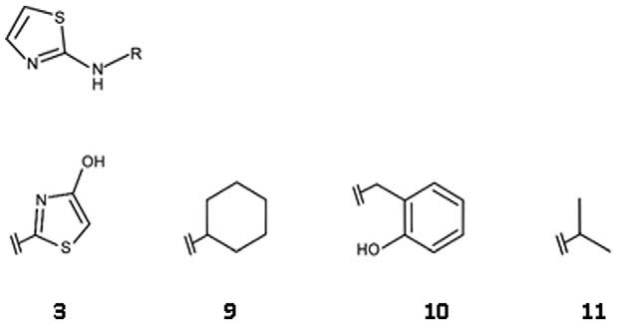
Chemical structures for analogues of ligand 3.

In the predicted binding mode, compound **3** is positioned between Tyr24 and Tyr175 and forms three hydrogen bonds to His25 in the cytidine binding pocket (one of them is a C-H•••N interaction similar to what is observed in serine/threonine protein kinases [44]) and an additional hydrogen bond to Asp130 (Figure 10a). Three commercially available analogues with the same core fragment predicted to interact with His25 but with different substituents on the amino group were selected for testing against *Ec*IspE (Figure 11, Table 3). All proved less potent than the screening hit. Compounds **9** (Figure 10b) and **11** (Figure 10d) are lacking a functional group that can interact with Asp130 and the hydroxyl group of **10** Figure 10c) is not in the right orientation required for a hydrogen bond with this amino acid. This might explain the loss in affinity of these ligands compared to the initial hit.

**Table 3:** Hit expansion for ligand 3.

ID	IC_50_ [M] (SD)*	Hill slope (SD)*	Ligand efficiency [kcal/mol per non-hydrogen atom]
**3**	1.6·10^−4^ (9·10^−6^)	1.0 (0.1)	0.50
**9**	9.4·10^−3^ (2·10^−3^)	1.4 (0.0)	0.30
**10**	6.6·10^−3^ (2·10^−4^)	1.1 (0.2)	0.27
**11**	2.0·10^−3^ (4·10^−4^)	1.1 (0.4)	0.50

For chemical structures see Figure 11.

* average values of three independent measurements, standard deviation in brackets.

**Figure 12:**
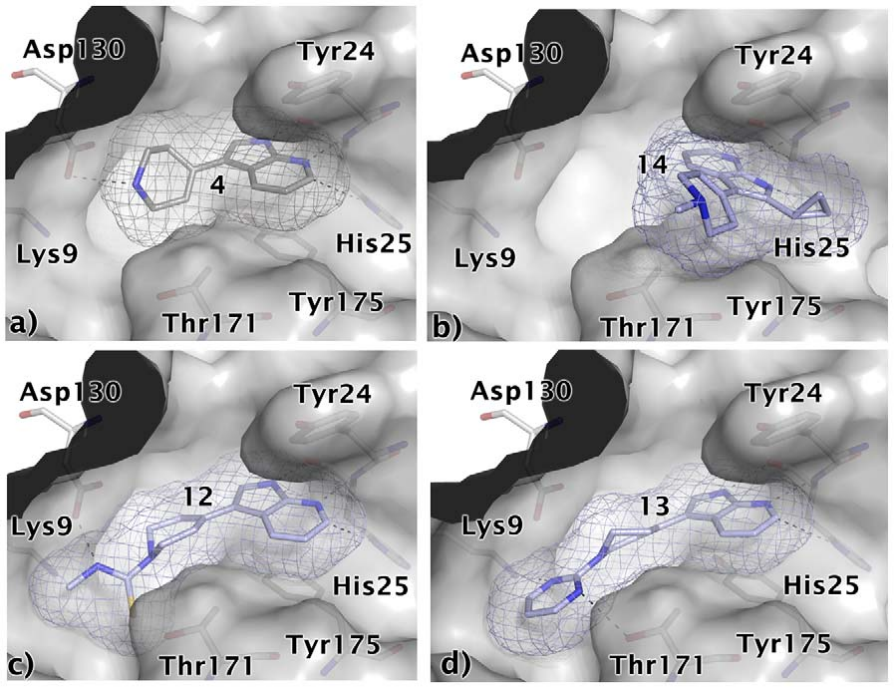
Modelled binding modes of compounds a) 4, b) 14, c) 12, and d) 13. The mesh represents the combined van der Waals radii of the ligand atoms. The pyrrolopyridine scaffold is predicted to be sandwiched between Try24 and Tyr175 and to form hydrogen bonds with His25. The virtual screening hit **4** is expected to form an additional salt bridge with Asp130 while. **12** is expected to form hydrogen bonds with Lys9 and Thr191 and **13** with Asp130. Ligand **14** presumably adopts a different binding mode in which the core fragment is flipped by 180° but still forms hydrogen bonds with His25.

**Figure 13:**
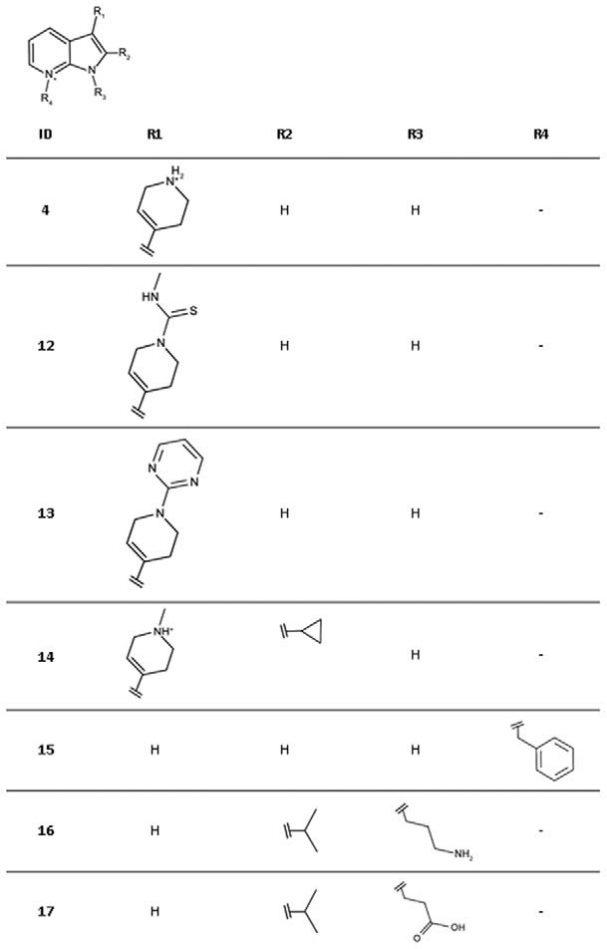
Chemical structures for analogues of ligand 4.

In the modelled binding mode of ligand **4** the pyrrolopyridine scaffold is sandwiched between Tyr24 and Tyr175 and potentially interacts with His25 via three hydrogen bonds (Figure 12a). In addition, the charged amino group in the tetrahydropyrimidinium ring may form a salt bridge interaction with Asp130. Commercially available analogues of this compound were selected to probe possible interactions with Asp130, His25 and the backbone of Lys145 (Figure 13, Table 4). Compounds **12** and **13** were chosen because of substituents on the tetrahydropyrimidinium ring which are likely to lower the *p*K_a_ resulting in neutral compounds under assay conditions. Accordingly, these compounds can no longer form a salt bridge with Asp130. In the predicted binding mode of **12** a neutral hydrogen-bond via the thio-urea group is formed with Asp130 instead while **13** is assumed to interact with Lys9 NZ and Thr171 OG1 (Figure 12c and d). Both compounds had slightly improved affinities compared to the screening hit suggesting that the additional interactions may compensate for the loss of the salt bridge. In order to accommodate **14** in the binding site in a similar binding mode as **4**, structural rearrangements are required to avoid a steric clash of the cyclo-propyl moiety with the backbone carbonyl group of Lys145 (Figure 2). An alternative binding mode for this compound is also possible in which the core is flipped by 180° compared to **4** but still forms hydrogen bonds with His25 (Figure 12b). In this orientation, both substituents are solvent exposed. Since this ligand had a 5.8-fold weaker IC_50_ value than the screening hit, either of the two alternatives appear to be less favourable than the interactions formed by the screening hit. Compounds **15–17** carry substitutions that prevent the same placement in the binding site with respect to His25 as suggested for the hit compound. None of these compounds displayed any inhibition of *Ec*IspE even when tested up to their solubility limit (1 to 5 mM) adding confidence to the proposed binding mode of **4**.

**Table 4 pone-0035792-t004:** Hit expansion for ligand 4.

ID	IC_50_ [M] (SD)*	Hill slope (SD)*	Ligand efficiency [kcal/mol per non-hydrogen atom]
**4**	1.5·10^−3^ (3·10^−4^)	1.3 (0.2)	0.32
**12**	8.3·10^−4^ (7·10^−5^)	1.0 (0.1)	0.27
**13**	4.7·10^−4^ (1·10^−4^)	1.0 (0.1)	0.26
**14**	8.7·10^−3^ (4·10^−4^)	1.0 (0.1)	0.19
**15**	n.i.^a^	-	-
**16**	n.i.		-
**17**	n.i.	-	-

For chemical structures see Figure 13.

* average values of three independent measurements, standard deviation in brackets.

a no inhibition at solubility limit measured.

Initially, we were unable to model plausible binding modes for the HTS hit compounds **7** and **8** (Table 2) in either the ATP or cytidine pocket. No analogues of **7** containing an indole moiety were present in the screening library. For compound **8**, 44 analogues with a quinazolinone core were found. Three of these showed >40% inhibition in the initial screens but such activity was not confirmed in the subsequent potency assay. Therefore, to establish initial SAR further analogues of the screening hits were identified using the similarity search method FTrees [45]. Our in-house library of commercially available compounds was screened using the HTS hits as query molecules and finally three analogues of **7** and ten of **8** were purchased for biochemical evaluation.

**Figure 14:**
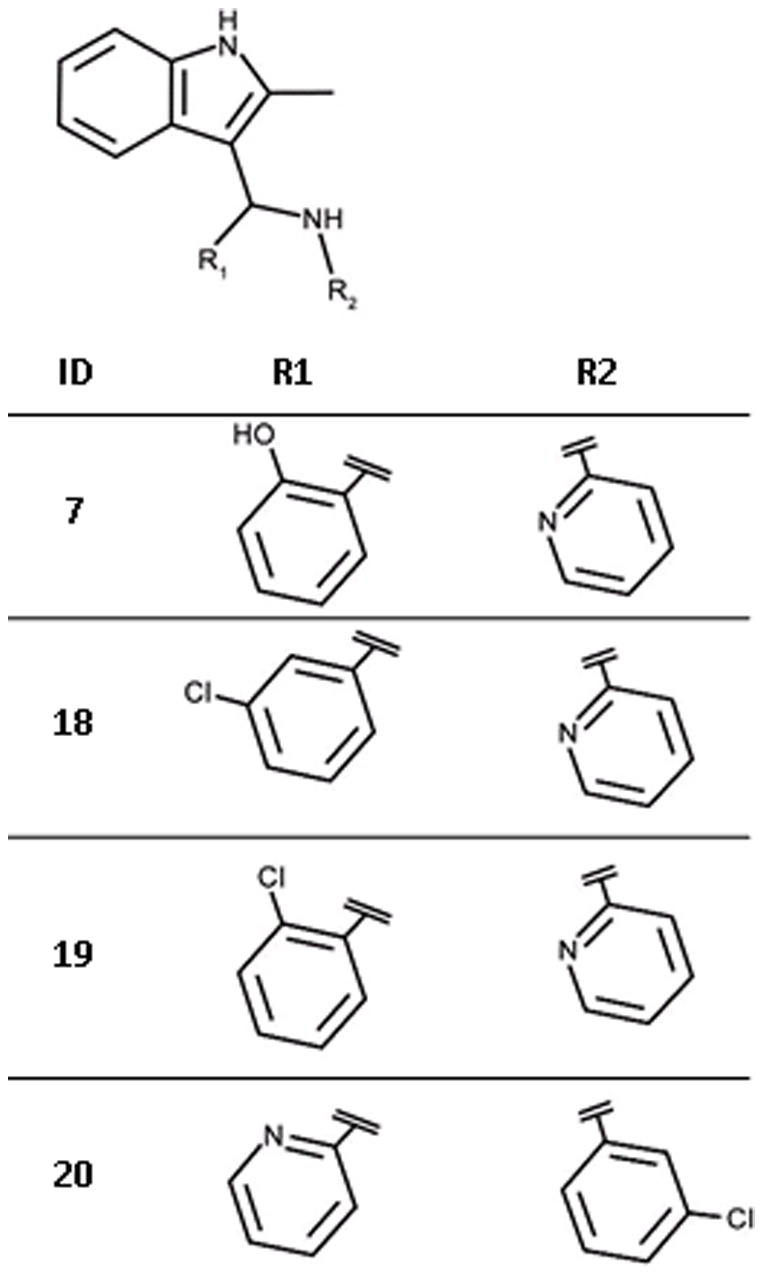
Chemical structures for analogues of ligand 7.

**Figure 15:**
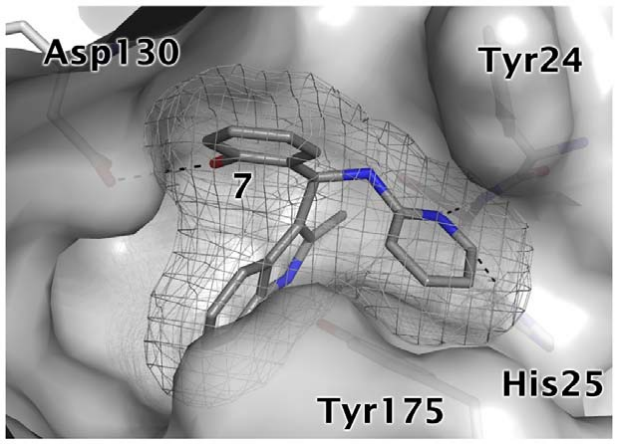
Modelled binding mode of compound 7. The mesh represents the combined van der Waals radii of the ligand atoms. In this orientation the pyridinyl group is stacked between Tyr24 and Tyr175 and forms hydrogen bonds with His25. Further, the hydroxyl group forms a hydrogen bond with Asp130 while the indolyl moiety is buried in a hydrophobic cleft.

In the case of compound **7**, the analogues displayed a one to two order loss in affinity for *Ec*IspE (Figure 14, Table 5). Common to all three analogues was the deletion of a hydroxyl group at R_1_ suggesting therefore that this group plays an important role for molecular recognition. Based on this observation, a potential binding mode for the *S* enantiomer of this compound which is a racemic mixture could be modelled in the cytidine pocket after manually adjusting some side chains (RMSD = 0.163 Å for relaxed side chain atoms compared to crystal structure used for docking). In the proposed pose, the pyridinyl substituent is stacked between the two aromatic residues in the cytidine binding site and additionally forms hydrogen bonds with His25 while the indolyl moiety is buried in a hydrophobic cleft (Figure 15). Further, the hydroxyl group of R_1_ is involved in a hydrogen bond with Asp130. The later interaction was already suggested to be important for binding of inhibitors **3** and **4** (Figure 10 and Figure 12). Consistent with this hypothesis, compounds **18** and **19**, which cannot form this interaction and, in the case of **19** would even lead to a steric clash with Asp130, displayed markedly reduced affinity compared to the screening hit **7**. Compound **20** bears a chlorophenyl group instead of the pyridinyl moiety and accordingly, favourable interactions with His25 are no longer possible. This is in agreement with the 130-fold reduced potency of this inhibitor compared to the hit compound.

**Table 5:** Hit expansion for ligand 7.

ID	IC_50_ [M] (SD)*	Hill slope (SD)*	Ligand efficiency [kcal/mol per non-hydrogen atom]
**7**	1.9·10^−5^ (2·10^−6^)	1.4 (0.1)	0.29
**18**	5.5·10^−4^ (5·10^−8^)	0.8 (0.0)	0.21
**19**	2.4·10^−3^ (4·10^−4^)	0.8 (0.1)	0.18
**20**	2.5·10^−3^ (3·10^−4^)	1.1 (0.1)	0.18

For chemical structures see Figure 14.

* average values of three independent measurements, standard deviation in brackets.

**Figure 16:**
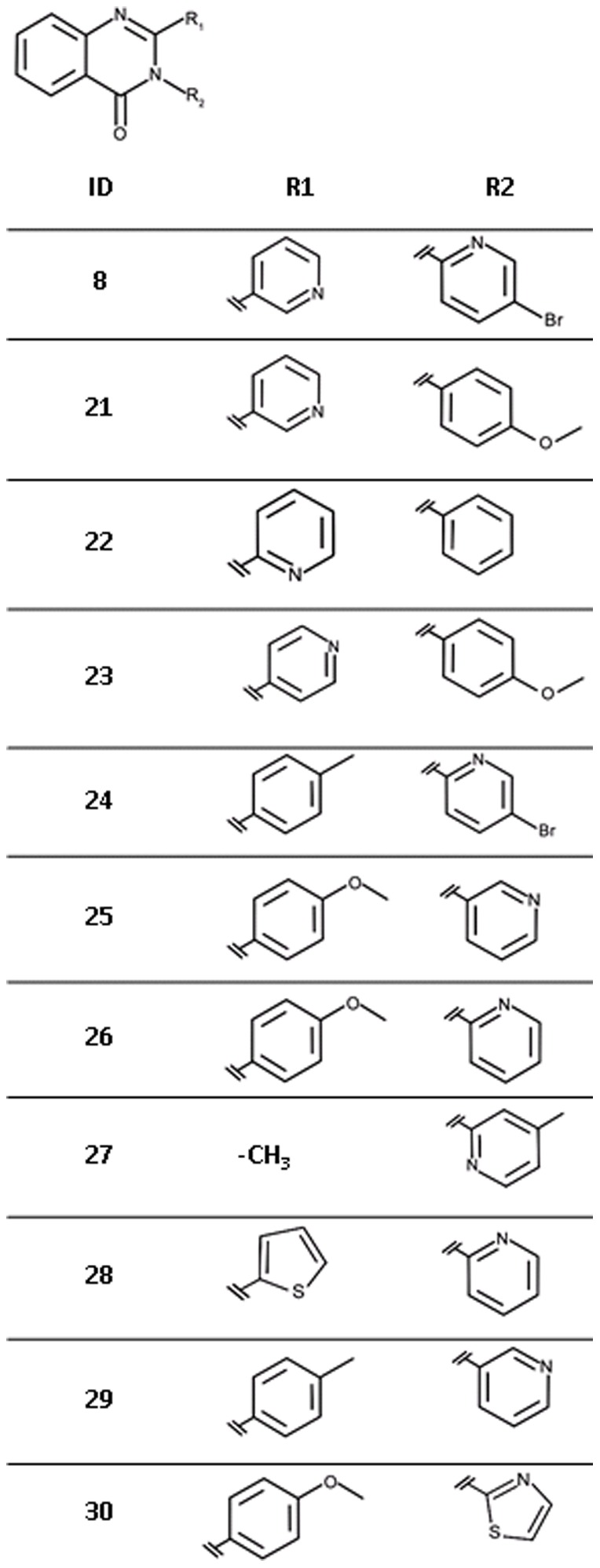
Chemical structures for analogues of ligand 8.

All purchased analogues of **8** proved less active than the screening hit (Figure 16, Table 6) and no plausible binding modes could be modelled for any of these compounds. Due to availability issues, most of the selected compounds contain more than one change compared to the hit compound or to each other therefore compromising the derivation of unambiguous SAR. However, it appears that a nitrogen atom at R_1_, preferably in the *meta* position, is beneficial for affinity (**21** vs. **23**). Replacement of the bromopyridinyl moiety of **8** with a methoxyphenyl group (**21**) is tolerated with a 11-fold loss in affinity. At this stage it is not possible to say if this is due to a loss of the hydrogen-bonding group, a steric clash or a combination of both.

**Table 6:** Hit expansion for ligand 8.

ID	IC_50_ [M] (SD)*	Hill slope (SD)*	Ligand efficiency [kcal/mol per non-hydrogen atom]
**8**	2.5·10^−6^ (4·10^−7^)	1.3 (0.1)	0.35
**21**	2.7·10^−5^ (9·10^−6^)	1.1 (0.3)	0.28
**22**	1.8·10^−4^ (4·10^−5^)	0.8 (0.1)	0.26
**23**	3.2·10^−4^ (8·10^−5^)	0.7 (0.1)	0.23
**24**	n.i.^a^	-	-
**25**	n.i.	-	-
**26**	n.i.	-	-
**27**	n.i.	-	-
**28**	n.i.	-	-
**29**	n.i.	-	-
**30**	n.i.	-	-

For chemical structures see Figure 16.

* average values of three independent measurements, standard deviation in brackets.

a no inhibition at solubility limit measured.

### Comparison of *in silico* and *in vitro* screening

Both, *in silico* and *in vitro* screening delivered two hits that were considered worth following up. Interestingly, the virtual screening and biochemical screening hits contained different chemical scaffolds. Aminothiazoles (Table 3) and pyrrolopyridines (Table 4) were only discovered using virtual screening while biochemical screening retrieved indole derivatives (Table 5) and quinazolinones (Table 6). The library used for HTS contained 185 aminothiazoles of which only 17 were unsubstituted in the 4-and 5-position of the thiazole moiety like the screening hit. None of them showed significant enzyme inhibition at 33 µM in the primary screen or could be confirmed in the secondary screen. Attempts to model the 17 unsubstituted analogues into the cytidine-binding pocket in a comparable binding mode to that predicted for **3** (Figure 10a) identified the following issues: (1) instead of an amine group some of these compounds contained an amide group of which the carbonyl oxygen would clash with the backbone carbonyl group of Lys145; (2) a large group attached to the amino group of the aminothiazole core would clash with Tyr175; (3) an acceptor functionality would be located too close to Asp130. These observations might explain why none of the compounds appeared as a hit in the biochemical screen. For compound **4**, only five analogues were present in the screening set. Two of them had the pyrrolo nitrogen position blocked which is believed to be essential for interaction with His25 (Figure 12) and one compound did not contain a substituent which allows interaction with Asp130. The remaining two compounds contained the tetrahydropyrimidinium moiety as present in the screening hit but again with a substitution that does not allow interaction with Asp130. All compounds showed <15% inhibition at the screening concentration of 33 µM. Compounds **7** and **8** were part of the initial virtual screening library but did not pass the first filter step as they violated the upper limit for number of heavy atoms and ring systems. For a retrospective docking exercise we therefore spiked the HTS library with ligands **3** and **4** docked all compounds into all four receptor setups (Table 7). While **3** and **4** ranked highly when the database was sorted by the score normalized for number of heavy atoms, **7** and **8** were not among the 5% top scoring compounds with either scoring scheme. This did also not improve when the receptor conformation that was manually adjusted to generate a binding mode for **7** (Figure 15) was used for docking (data not shown).

**Table 7:** Ranks for the *in silico* and *in vitro* screening hits when the HTS library is docked against *Aa*IspE without prior filtering for physico-chemical properties or pharmacophore constraints.

ID	Rank_total^1^	Rank_heav^2^
	(Setup^3^)	(Setup^3^)
**3**	3344	45
	(3)	(3)
**4**	36	17
	(4)	(4)
**7**	802	3782
	(3)	(3)
**8**	532	3313
	(1)	(1)

For chemical structures see Figure 7 and Figure 9.

1 using the total score of docking for ranking.

2 using the score divided by the number of heavy atoms of the molecule for ranking.

3 setup 1: His25 protonated at ND, no ADP present; setup 2: His25 protonated at ND, ADP present; setup 3: His25 protonated at NE, no ADP present; setup 4: His25 protonated at NE, ADP present.

## Discussion

IspE is a potential target for new antimicrobials for a range of pathogens [10], [11], [12], [13], [14], [15]. Through a combination of virtual and biochemical screening four new inhibitors for this enzyme were discovered (Table 1 and Table 2). They show IC_50_ values of 2.5 µM (**8**), 19 µM (**7**), 160 µM (**3**), and 1.5 mM (**4**), respectively and ligand efficiencies of 0.29 kcal/mol per non-hydrogen atom or better. The inhibitors do not resemble any previously known IspE inhibitors (Figure 4). [16], [17], [18], [23], [24]. The physico-chemical properties of the virtual screening hits are in the fragment-like space (MW<300 Da, clogP≤3, number of hydrogen-bond donors ≤3 and hydrogen-bond acceptors ≤6) while those of the HTS hits are in the lead-like space (MW<400, clogP≤4, number of hydrogen-bond donors ≤4 and hydrogen-bond acceptors ≤8) rendering these new ligands promising starting points for drug discovery (Figure 4).

Unfortunately, co-crystallisation of the screening hits with *Aa*IspE was not successful. This might be related to solubility issues and, in the case of **8**, conformational changes requiring new crystal forms since the crystals dissolved when the compound was added. However, for three of the four screening hits and their analogues putative binding modes could be modelled (Figure 10, Figure 12, and Figure 15). In the suggested binding modes, the ligands bind into the cytidine pocket. They form π-stacking interactions with Tyr24 and Tyr175 and hydrogen bonds with His25 and Asp130. These binding modes are consistent with SAR derived from analogues indicating that disrupting interactions with His25 or Asp130 leads to a drop in binding affinity (Table 3–[Table pone-0035792-t004] Table 5, Figure 10, Figure 12, and Figure 15). However, due to availability issues more subtle changes in the compounds could not be probed. Therefore, SAR remains tentative. For a more extended chemical evaluation and to increase potency synthetic efforts around the retrieved hits are required.

We decided to adopt a virtual screening cascade with a series of increasingly stricter filter steps (Figure 5). The aim of this strategy was to early remove compounds that were not attractive starting points for drug discovery and had no potential to bind to the cytidine binding site of IspE. This made the process faster but also easier to mange as we had to deal with a smaller number compounds for docking. Further, molecular docking can result in poses in which polar groups of the ligands do not form hydrogen-bonding interactions with the receptor or vice versa and are therefore likely to be false positive predictions [35]. These can often be removed by using a pharmacophore to filter the docking solutions and such improve the results [27], [46]. Therefore, all docking poses were post processed. The successful application of similar strategies to other targets gave us confidence in this approach [47], [48]. To consider the presence and absence of the co-factor and the potential tautomers of His25 (Figure 6), four different setups for docking were prepared. While compounds from all setups were chosen for testing, for the most promising hit compounds (**3** and **4**) only one of the possible tautomers for His25 was found to be important (Table 1). In this representation, a protonated NE of His25 is required, which is different from the substrate-bound state of the pocket (Figure 2). Coincidently, this is the same tautomer that was used for modelling the binding mode of the biochemical screening hit **7** (Figure 15). The virtual screening library contained a mix of fragment- and lead-like compounds. To favour compounds that were predicted to bind with high ligand efficiency we normalized the scores by the number of heavy atoms. Both, compounds with a high total score and a high normalized score were carried forward for visual inspection. Interestingly, all compounds that showed any IspE inhibition (Table 1) were selected based on the latter criteria and were in the fragment-like space making this exercise yet another success story of fragment-based virtual screening [25], [47], [48], [49], [50], [51], [52].

*In silico* and *in vitro* screening retrieved chemically distinct hits. (Table 1 and Table 2). On the basis of structural considerations and for reasons of cost efficiency, it was decided to use a small, focussed library containing about 6,000 compounds for *in vitro* screening. Despite the limited size, the scaffolds of both virtual screening hits were contained in this library. With just five examples, chemical space around hit **4** was poorly represented. It is therefore unsurprising, that this compound class was not retrieved using *in vitro* screening. In contrast, 185 compounds containing aminothiazoles were part of the screening library yet this compound class did not appear among the HTS hits. A reason for this might be that only 17 aminothiazoles were unsubstituted in the 4- and 5-position as in the screening hit and all of them had additional functionalities that were predicted to lead to a steric clash in the binding site and/or unfavourable interactions with Asp130. It is an on-going debate as to how many analogues should be contained in a screening library to have a good chance to discover a hit. Often, 50–100 analogues are considered sufficient [40], [53], [54]. Clearly, that was not the case in our investigation. Given the appropriate infrastructure, large libraries can be screened *in silico* in a cost efficient manner, overcoming a problem with *in vitro* screening of having to preselect library compounds and thus to restrict commercially available chemical space. However, it is well known that docking performance decreases with increasing molecular size and number of rotational bonds [55], [56], [57]. Therefore, the complexity of the compounds in the *in silico* library was limited (Figure 5). As a consequence, the HTS hits **7** and **8** were rejected as they violated the upper limit of number of heavy atoms (not more than 23) and ring systems (not more than two). Even if the HTS library had been used for virtual screening, **7** and **8** could not have been discovered (Table 7). Both compounds ranked poorly when docking this library against IspE and more promising compounds like **3** and **4** would still have been favoured for biochemical testing. It remains unclear which binding mode **8** adopts when binding to IspE and therefore why docking failed. In contrast, we speculated that binding of **7** requires a conformational change of the receptor (Figure 15). When this receptor conformation was used for docking, a more sensible binding mode was obtained but ranking was still poor. This points to a limitations of molecular docking: While progress has been made in considering receptor flexibility in practice, it is still often neglected when screening large databases due to speed issues, scoring problems and difficulties in predicting relevant protein conformations [58]. As a result, ligands that require a conformational change of the receptor in order to bind will not be retrieved. Furthermore, fragment hits are often weaker ligands than the larger HTS hits [25]. This was also the case here. While the HTS hits showed affinities in the low micromolar range, the virtual screening hits were less potent with IC_50_ values in the high micromolar to low millimolar range (Table 1 and Table 2). However, the ligand efficiencies of the virtual screening hits were comparable or higher than those of the HTS hits. Assuming that the ligand efficiency stays approximately constant during optimisation [59], despite their weaker potencies the virtual screening hits are therefore at least as good starting points for a hit-to-lead program as are the HTS hits. A benefit of the virtual screening hits was that they came immediately with a hypothesis about which binding mode they might adopt. This allowed rational selection of analogues to probe the binding mode and derive SAR. In contrast, for one of the HTS hits (**7**) a binding mode could only be suggested after derivatives selected using ligand-based similarity screening were tested. For inhibitor **8**, even this approach did not lead to a binding hypothesis. Finally, retrieval of the virtual screening hits was a prerequisite to conduct a robust HTS. Since none of the standard kinase inhibitors turned out to be active against IspE and previously known IspE inhibitors were not commercially available, the virtual screening hits served as quality control standards for biochemical screening ensuring that our screening results were reliable [60].

### Conclusions

The DOXP pathway is an essential aspect of metabolism and a validated target for antimicrobials for a range of pathogens. A combination of *in silico* and *in vitro* screening against IspE, the fourth enzyme in this pathway, has identified non-substrate like inhibitors. The two strategies were complementary, delivering chemical distinct hits. However, running a robust and reliable biochemical screening campaign only became possible after the virtual screening hits were identified since no commercially available inhibitors for IspE which could serve as quality control standard were known. Four of the identified hits were followed-up with analogues. While most of the commercially available analogues were less potent than the screening hits, they allowed SAR to be established and identification of crucial amino acids for ligand binding. The new inhibitors possess favourable physico-chemical properties and good ligand efficiencies. They therefore constitute promising starting points for further optimization.

## Methods

### Computational methods

Figures of protein-ligand complexes were prepared using PyMol (Open-Source PyMOL 0.99rc6, Copyright 2006, DeLano Scientific LLC).

#### Structure-based virtual screening

Our in-house MySQL library of commercially available compounds [40] was filtered for compounds fulfilling the following criteria: between one and five hydrogen-bond donors, one and ten hydrogen-bond acceptors, between nine and 23 heavy atoms, clogP between −1 and 4, less than seven rotatable bonds, total charge between −1 and +1, and one or two ring systems (fused rings were counted as one ring system). In addition, compounds containing potentially reactive or toxic functionalities [40] were rejected.

Unity from the Sybyl package (Tripos A. St. Louis, MO, U.S.A) was used for pharmacophore filtering. Pharmacophoric points were defined protein based (Protein Data Bank (PDB) code 2v8p) with default settings to include the desired directionalities for hydrogen bonding. The initial pharmacophore search was performed with flexible ligand molecules, allowing rotation and conformational changes to match the required features. At least two of the possible four features had to be fulfilled to pass this filter. For filtering the docking poses, the docked ligands were kept rigid and no translations and rotations were allowed.

A database containing all compounds passing the pharmacophore filter step in a format suitable for docking and considering multiple protonation states and tautomers was prepared as described previously [47].

The *Aa*IspE crystal structure (PDB code 2v8p) was the receptor for docking. Four different setups were prepared taking into account the possible tautomers of His25 (Figure 6), and the presence or absence of the co-factor. Polar hydrogen atoms were added to the receptor and their positions minimised using the MAB force field [61] as implemented in MOLOC (Gerber Molecular Design: Switzerland). Partial charges for the co-factor were calculated using AMSOL [62]. Spheres as matching points for docking were placed around the cytidine heterocycle of the bound substrate. The sphere set defining the buried region of the binding site was generated around the whole substrate and co-factor (if present in the setup). Grids to store information about excluded volumes, electrostatic and van der Waals potential, and solvent occlusion were calculated as described earlier [31]. DOCK 3.5.54 [63], [64] was used to dock the molecules into the binding sites. The following settings were chosen to sample ligand orientations: ligand and receptor bins were set to 0.5 Å, and overlap bins were set to 0.4 Å; and the distance tolerance for matching ligand atoms to receptor matching sites ranged from 1.1 to 1.2 Å. Each docking pose which did not place any atoms in areas occupied by the receptor was scored for electrostatic and van der Waals complementarity and penalised according to its estimated partial desolvation energy [65]. The docking setup was validated by successful predictions of the binding modes of CDP, CDP-ME, and cytosine (data not shown). For each compound in the screening database, only the best-scoring representation (tautomer, protonation state, multiple ring alignment) was stored in the final docking hit list.

#### Manual modelling of binding modes

The binding mode for compound **7** was modelled manually using MOLOC. The ligand was placed in possible orientations in the binding site and the positions were minimised allowing side chains to relax while backbone atoms were kept rigid. The most convincing mode was selected based on complementarity of functional groups between ligand and receptor and the avoidance of van der Waals clashes as well as accordance with the SAR hypothesis of this compound series.

For analogues of compounds **3** and **4**, the core fragment was initially superposed on the docking poses of the parent compounds. Manual adjustment of additional functional groups was followed by minimization with the MAB force field as implemented in MOLOC whereas the ligand was kept flexible and the binding pocket rigid.

#### Feature Trees search

Molecular similarity searches were performed with the program Feature Trees, version 2 (BiosolveIT, Germany) [45]. Compounds **7** and **8** were used as query molecules. The ligands were pre-processed with Sybyl as follows: atom types and formal charges were assigned, hydrogen atoms were added, 3D structures were generated and energy minimised. Our in-house compound database [40] was converted to Feature Trees and used for searching similar compounds to the query molecules. Similarity values between the database compounds and the query molecules at “level 0” (global similarity considering all features in each Feature Tree at once) and similarity values at “level x” (best similarity of two Feature Trees after a recursive comparison algorithms has been used) were calculated using the match search algorithm. Default values were assigned to all adjustable parameters. Hits with a similarity value of above 0.95 were inspected to shortlist compounds for purchase and testing.

### Biochemical methods

#### Protein preparation

Recombinant *Ec*IspE was purified following published methods [18], [19]. For each batch of enzyme the kinetic constants *K*_M_ and *k*_cat_ were determined using an established assay [18] and compared with published data as a quality control measure.

#### IspE inhibition assay

Inhibition of *Ec*IspE was measured using the Promega Kinase-Glo®-Plus kit [66]. This kit determines the remaining ATP concentration after the kinase reaction took place by converting luciferin to oxyluciferin via an ATP-dependent luciferase. Oxyluciferin is chemoluminescent and can be detected using a plate reader.

The assay was performed under the following conditions: 90 µM ATP, 500 µM CDP-ME, 200 nM *Ec*IspE in 100 mM Tris-HCl, pH 8.5, 20 mM MgCl_2_, 2% DMSO, 0.01% (v/v) Triton-X 100. The reaction was carried out in 384-well plates with 25 µl reaction volumes, incubated at 25°C for 180 min, and stopped with 25 µl Kinase-Glo® Plus reagent (Promega, Madison, USA). The chemoluminescence signal was developed for 45 min at room temperature and read out in a plate reader (TopCount, PerkinElmer, USA). To monitor assay performance and to control for inhibition of the luciferase of the Kinase-Glo® Plus kit wells containing either the reaction mixture plus compound **3** or **4** or all ingredients except for CDP-ME were also prepared. Further, as quality control for each assay plate, wells containing the kinase reaction without an inhibitor, and wells containing only ATP in assay buffer were prepared. The signals of these wells were averaged (AV_HIGH_, AV_LOW_) and used to calculate the signal to noise ratio (AV_HIGH_/AV_LOW_) and Z′ (1−
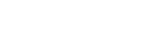
; SD: standard deviation). The best performance was obtained with an ATP concentration of 90 µM and a CDP-ME concentration of 500 µM (Signal to noise ratio = 3.3, Z′ = 0.69 in assay development). In this setup the ATP and CDP-ME concentrations are ∼0.2×*K*_M_ and ∼3×*K*_M_, respectively [16]. Consequently, the assay can be used to detect ligands binding to both the substrate and co-factor binding sites.

#### IC_50_ determinations and ligand efficiencies

Compound **1** was ordered from Maybridge (Fisher Scientific UK Ltd, Leicestershire, UK), compounds **2** and **3** from ChemDiv (San Diego, USA), compounds **4**, **7**, **9**, **11**, **12**, **13** from Enamine Ltd. (Kiev, Ukraine), compounds **5** and **6** from Sigma-Aldrich Chemie GmbH (Munich, Germany), compound **8** from TimTec LLC (Newark, USA), compounds **10**, **14**, **16**, **17** from Asinex Ltd. (Moscow, Russia), compound **15** from Bionet (Key Organics, Cornwall, UK), compounds **18**, **19**, **20** from Princeton BioMolecular Research Inc. (NJ, USA), compound **21** from ChemBridge Corporation (San Diego, USA), and compounds **22** and **23** from Aurora Fine Chemicals LLC (San Diego, USA).

Identity and purity of key compounds (**3**, **4**, **7–14**, **18**, **19**, and **21**) were analysed by LC-MS using a Bruker MicroTof mass spectrometer coupled to an Agilent HPLC 1100, with a diode array detector in series. The column used was a Phenomenex Gemini C18 column, 50×3.0 mm, 5 mm particle size. The following method was used: mobile phase, water/acetonitrile+0.1% HCOOH 80∶20 to 5∶95 gradient over 3.5 min, and then held at for 1.5 min; flow rate 0.5 mL/min. All investigated compounds had the correct identity judged by the M+ data. Compound **12** was 70% pure, compounds **4**, **7**, **8**, **19** and **21** between 81 and 86% and the remaining compounds >95%.

Compounds were dissolved in DMSO at concentrations between 10 and 200 mM (depending on maximal solubility) and added to the reaction mixture with a final concentration of 2% DMSO (v/v). For compounds where IspE inhibition was indicated, a 1∶3 dilution series in DMSO was prepared and tested in the same endpoint assay. IC_50_ values were derived by non-linear regression with a 4-parameter fit to the following equation in GraFit (Erithacus Software Ltd., Surrey, UK).
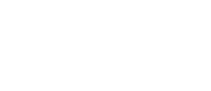
where y: measured signal of assay: y_max_: saturated signal: [I]: inhibitor concentration: slope: Hill slope; y_min_: background signal of assay.

As the assay was performed at relatively high substrate concentrations (∼3×*K*_M_) for ligand efficiency calculations [39], the measured IC_50_ values were first converted to *K*_i_ values. Assuming competitive inhibition of the cytidine binding site the following equation was used:
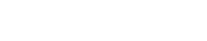
where K_M_ is 150 µM and [CPD-ME] = 500 µM [16].

Ligand efficiencies were calculated using the formula:
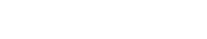
where T = 298 K and R = the ideal gas constant.

#### *In vitro* screening of the kinase library

Compounds from the kinase set, stored as 3.3 mM stock solutions in DMSO, were dispensed into black 384 well assay plates (Thermo Scientific Matrix,) using a HummingBird™ (Cartesian,) with a 250 nl dispenser head. 15 µl of the enzyme- and substrate solution were dispensed into each well with a PlateMate Plus (Thermo Scientific Matrix,). The reaction was started with 10 µl ATP solution dispensed by a WellMate robot (Thermo Scientific Matrix,).
